# Correlation between family history and characteristics of breast cancer

**DOI:** 10.1038/s41598-021-85899-8

**Published:** 2021-03-18

**Authors:** Lei Liu, Xiaomeng Hao, Zian Song, Xiangcheng Zhi, Sheng Zhang, Jin Zhang

**Affiliations:** 1 The Third Department of Breast Cancer, China Tianjin Breast Cancer Prevention, Treatment and Research Center, Tianjin Medical University Cancer Institute and Hospital, National Clinical Research Center of Cancer, Huanhu West Road, Tianjin, 300000 China; 2 Key Laboratory of Cancer Prevention and Therapy, Tianjin, China; 3 Tianjin’s Clinical Research Center for Cancer, Tianjin, China; 4 Key Laboratory of Breast Cancer Prevention and Therapy, Tianjin Medical University, Ministry of Education, Tianjin, 300000 China

**Keywords:** Oncology, Cancer, Breast cancer

## Abstract

Family history is a major risk factor for breast cancer; approximately 5–10% cases of breast cancer are associated with a family history. Herein, we investigated the link between family history and breast cancer features to elucidate the importance of family history in the diagnosis and treatment of breast cancer. Data from 10,549 patients with breast cancer were collected from 2014 to 2017. Detailed information about the family history of the patients including the degree and number of relatives affected and the types of cancer was recorded. The tumors were pathologically and clinically classified based on the stage, grade, ER, PR, HER2, Ki-67 status, and subtypes, according to standard guidelines. Data were analyzed using χ^2^ test and multiple logistic regression. Patients with a family history of other cancer types were significantly older at diagnosis than patients with a family history of breast/ovarian cancer (*p* = 0.002) and those without a family history of cancer (*p* < 0.001). Patients without a family history of cancer were typically diagnosed at a later stage, including high frequency in N2 (*p* = 0.035) and TNM stage III (*p* = 0.015). Compared with patients with second-/third-degree relatives, those with first-degree relatives having breast/ovarian cancer had a higher median age (54.1, *p* < 0.001) at diagnosis and showed more advanced disease. No significant difference was found between ER, PR, and HER2 status in patients with and without a family history of cancer. Family history of breast cancer can influence the cancer characteristics of the patients at diagnosis, especially patient age, tumor stage, and grade.

## Introduction

Breast cancer is the most common type of cancer among women worldwide, and it seriously affects their health. According to reports from World Health Organization, approximately 2.1 million new cases of breast cancer were diagnosed globally in 2018, and breast cancer is the leading cause of cancer-associated deaths in women^1,2^. Many factors, including environmental and genetic factors, contribute to the development of breast cancer, including but not limited to age, diet, body mass index (BMI), reproductive history, common oncogenes, breast density, and family history^3^. Family history is one of the most well-known factors that have a major impact on breast cancer risk, with an odds ratio of 1.71 (95% CI 1.59–1.84)^3^. Another study using a large patient cohort showed that women with two or more relatives having a history of breast cancer have a 2.5-fold (95% CI 1.83–3.47) increased risk of developing breast cancer^4^. All these data suggest that family history of breast cancer should be treated as one of the most important screening factors in breast cancer prevention.

Breast cancer and ovarian cancer are female hormone-responsive cancers that are closely linked with each other^5^. Approximately 5–10% of newly diagnosed patients with breast cancer have a family history of breast cancer or ovarian cancer^6^, suggesting the role of genetic or non-genetic inheritance in the development of breast cancer. Several well-known genes have been shown to be enriched in families with breast or ovarian cancers. For instance, 50–85% of women with mutations of breast cancer type 1 and type 2 genes (BRCA1 and BRCA2) will develop breast and/or ovarian cancer in their lifetime^7^. Breast/ovarian cancers caused by mutations in these common genes are defined as Hereditary Breast and Ovarian Cancer (HBOC), as mutations can be inherited in family members and cancers are enriched in these families. The risk of developing other types of cancer also increases with these gene mutations, including fallopian tube cancer, pancreatic cancer, and acute myeloid lymphoma^7,8^. Meanwhile, some patients with a family history of breast or ovarian cancer do not have these common genetic mutations. Other uncommon mutations including PTEN and ATM can also contribute to the development of breast and ovarian cancer^9,10^. Owing to the high risk of breast and ovarian cancer in patients with these mutations, breast self-examination was recommended for women with these mutations who are aged over 18 years, and clinical breast examination was offered from the age of 25 years, as against the usual age of 40 years recommended for clinical breast examination for women without these mutations, according to the National Comprehensive Cancer Network (NCCN) guidelines^11,12^.

However, not all patients with a family history of breast cancer have these common or uncommon mutations. This could be attributed to the fact that family history can impact many factors that could contribute to the development of breast cancer. Some critical factors that affect breast cancer risk, such as breast density and BMI, also show familial aggregation^13,14^. This could be the result of both genetic inheritance and lifestyle similarities between family members. These facts suggest that family history is a comprehensive risk factor for breast cancer, and a more detailed assessment of family history could have major clinical significance in this regard.

The overall risk of breast cancer increases with closer relatives developing breast/ovarian cancer at a younger age. Even in women without common mutations, the 10-year absolute risk of developing breast cancer reaches 14.1% if they have first-degree relatives who developed breast cancer before the age of 40 years. The risk is even higher when women are positive for BRCA1/2 or other common genetical mutations^15^. As genetic testing is still uncommon in the general population, clinical consultation is the fundamental method for understanding the importance of the correlation between family history and the risk of developing breast cancer. A detailed family history of cancer including the degree of relatives, tumor types, age at diagnosis, and age at death should be taken at the patient’s diagnosis.

The role of family history in the risk of developing breast cancer is well studied. However, the relationship between breast cancer progression and family history is unknown. Breast cancer progression is affected by multiple factors, including but not limited to subtypes (estrogen receptor [ER], progesterone receptor^3^, and human epidermal growth factor receptor 2 [HER2] status), grade, and stages. The link between family history and the clinical and pathological features of breast cancer at diagnosis has been tested in several studies; however, no clear conclusion has been drawn^7,16^. As family history plays a critical role in the development of breast cancer, investigating the relationship between family history and breast cancer characteristics may help predict the progression of breast cancer and guide treatment strategies.

## Results

### Pathological and clinical summary of patients

From March 2014 to July 2017, a total of 10,549 patients with breast cancer were treated in the Department of Breast Surgery, Tianjin Medical University Cancer Institute & Hospital. The mean age of the patients was 52.7 (range 18–89) years. The primary tumors of most of the patients were classified as T2 (2 cm < greatest dimension ≤ 5 cm), which accounted for 57.8% of the cohort. About 60.7% of the patients were negative for lymph node metastasis at diagnosis. Thus, the majority of the patients in our cohort were classified as stage II, based on the TNM staging system. The tumors of 75.9% of the patients were classified as histologic grade II. About 74.4% of patients tested positive for ER/PR, 14.1% of patients tested positive for HER2, and 63.5% of patients had high levels of Ki-67. About 23.1% of patients in our cohort were classified as having luminal A subtype breast cancer; 51.3%, as having luminal B subtype; 8.0%, as having HER2 subtype; and 17.6%, as having TNBC subtype. In general, we had a balanced patient cohort, the majority of whom had early-stage disease. The detailed patient information is shown in Table 1.

**Table 1:** Basic patient information.

	Number of patients	Percentage
**Age at diagnosis (years)**		
Age range	18–89	
Median age	52.7	
**Primary tumor stage**		
T0	765	7.3
T1	2842	26.9
T2	6099	57.8
T3	691	6.6
T4	152	1.4
**Lymph node stage**		
N0	6406	60.7
N1	2499	23.7
N2	880	8.3
N3	764	7.2
**TNM stage**		
0	761	7.2
I	2039	19.3
II	5776	54.8
III	1949	18.5
IV	24	0.2
**Histologic grade**		
I	370	3.5
II	8009	75.9
III	2170	20.6
**ER/PR status**		
Positive	7848	74.5
Negative	2692	25.5
**HER2 status**		
Positive	1487	14.1
Negative	9062	85.9
**Ki-67 status**		
High	6703	63.5
Low	3846	36.5
**Subtypes**		
Luminal A	2433	23.1
Luminal B	5415	51.3
HER2	847	8.0
TNBC	1854	17.6

### Regional effects on family history of breast cancer

Of a total of 10,549 patients in our cohort, 5370 patients were from coastal cities and 5179 patients were from non-coastal cities. In total, 431 patients (8.0%) from coastal cities had a family history of breast/ovarian cancer, which was significantly higher than the percentage of patients who had a family history of breast/ovarian cancer in non-coastal cities (345 cases, 6.7%, *p* = 0.007). Further, the rate of patients with a family history of other types of cancer was also significantly higher in coastal cities (1123 cases vs. 916 cases, 20.9% vs. 17.7%, *p* < 0.001) The detailed data are shown in Table 2. These data suggest a regional effect of the family history of cancer, with a more enriched family history observed in patients from coastal cities.

**Table 2:** Regional effects on family history of breast cancer.

	Coastal cities (n = 5370)		non-coastal cities (n = 5179)		*p*
	n	%	n	%	
Family history of breast/ovarian cancer	431	8.0	345	6.7	0.007
Family history of other cancer	1123	20.9	916	17.7	< 0.001
No family history of cancer	3816	71.1	3918	75.7	< 0.001

### Family history, and pathological and clinical features of breast cancer

Among all the patients in our cohort, 776 patients had a family history of breast/ovarian cancer, which accounted for 7.4% of the entire patient cohort, a finding that was consistent with those of other studies^4,6^. Of the 776 patients with a family history of breast/ovarian cancer, 744 patients (95.9%) had a family history of breast cancer only, 24 patients (3.1%) had a family history of ovarian cancer only, and 8 patients (1.0%) had a family history of both breast and ovarian cancer. In total, 2039 patients (19.3%) in our cohort had no family history of breast cancer but had other types of cancer, and 7734 patients (73.3%) had no family history of cancer. Patients were grouped based on a family history of breast/ovarian cancer (FHBO), family history of other cancer (FHO), and no family history of cancer (non-FH), and the pathological and clinical features of breast cancer were compared between these three groups. The median age of patients in the FHO group was 54.1 years, which was significantly higher than that of patients in the FHBO (52.5 years) and non-FH (52.3 years) groups. No significant difference in age was found between patients in FHBO and non-FH groups. Histological grading showed more patients with grade I in non-FH group than in FHBO and FHO groups (*p* = 0.019). As the majority of patients were negative for lymph node metastasis, similar findings were observed in all three groups. Approximately 60% of patients in all three groups were classified as stage N0 (no tumor in axillary and nearby lymph nodes). Significant differences between groups were observed in the percentage of patients classified as stage N2 (4–9 axillary lymph nodes or internal mammary lymph nodes positive for tumor). Fewer patients in the FHBO group (6.8%) were classified as N2 stage, compared with the FHO (7.3%) and non-FH groups (8.8%). Further, based on the TMN staging system, patients in non-FH group (19.2%) had a significantly high proportion of stage III cancer, compared with patients in the FHBO (15.7%) and FHO (16.8%) groups. These data suggest a lower stage of cancer at diagnosis among patients who had a family history of breast/ovarian cancer and other types of cancer, compared with patients without a family history of cancer. The ER, PR, and HER2 statuses of the patients’ tumors were evaluated, and no significant difference was observed between these three groups. However, the percent of luminal B subtype of breast cancer was slightly higher in the non-FH group (51.9%) than in the other groups (50.6% and 49.3%, *p* = 0.037). Detailed analysis is shown in Table 3.

**Table 3:** Family history of cancer and patients’ tumor characteristics.

	Family history of breast/ovarian cancer (n = 776)		Family history of other cancer (n = 2039)		No family history of cancer (n = 7734)		*p* 1*	*p*2*	*p*3*
	N	%	N	%	N	%			
**Age at diagnosis (years)**									
Age range	23–88		20–89		18–89		0.002^a^	0.342	< 0.001^a^
Median age	52.5		54.1		52.3				
**Primary tumor stage**									
T0	61	7.9	154	7.6	550	7.1	0.783	0.441	0.493
T1	221	28.5	524	25.7	2097	27.1	0.135	0.415	0.199
T2	429	55.3	1207	59.2	4463	57.7	0.060	0.193	0.225
T3	54	7.0	131	6.4	506	6.5	0.609	0.656	0.848
T4	11	1.4	23	1.1	118	1.5	0.530	0.814	0.180
**Lymph node stage**									
N0	495	63.8	1244	61.0	4667	60.3	0.175	0.061	0.584
N1	178	22.9	498	24.4	1823	23.6	0.410	0.692	0.421
N2	53	6.8	149	7.3	678	8.8	0.661	0.066	0.035
N3	50	6.4	148	7.3	566	7.3	0.450	0.370	0.926
**TNM stage**									
0	61	7.9	154	7.6	546	7.1	0.783	0.408	0.443
I	158	20.4	395	19.4	1486	19.2	0.555	0.440	0.872
II	433	55.8	1142	56.0	4201	54.3	0.921	0.430	0.173
III	122	15.7	343	16.8	1484	19.2	0.482	0.019	0.015
IV	2	0.3	5	0.2	17	0.2	1.000	0.690	0.794
**Histologic grade**									
I	23	3.0	55	2.7	292	3.8	0.700	0.254	0.019
II	575	74.1	1576	77.3	5858	75.7	0.074	0.309	0.145
III	178	22.9	408	20.0	1584	20.5	0.087	0.107	0.638
**ER/PR status**									
Positive	566	72.9	1503	73.7	5779	74.7	0.677	0.277	0.352
Negative	210	27.1	536	26.3	1955	25.3			
**HER2 status**									
Positive	97	12.5	288	14.1	1102	14.2	0.262	0.182	0.886
Negative	679	87.5	1751	85.9	6632	85.8			
**Ki-67 status**									
High	505	65.1	1290	63.3	4908	63.5	0.372	0.372	0.872
Low	271	34.9	749	36.7	2826	36.5			
**Subtypes**									
Luminal A	173	22.3	497	24.4	1763	22.8	0.247	0.751	0.132
Luminal B	393	50.6	1006	49.3	4016	51.9	0.536	0.496	0.037
HER2	56	7.2	170	8.3	621	8.0	0.328	0.425	0.650
TNBC	154	19.8	366	17.9	1334	17.2	0.247	0.069	0.457

^a^*p* < 0.05 with Tamhane T2 correction. *p1: FHBO vs. FHO. *p*2: FHBO vs. non-FH. *p*3: FHO vs. non-FH.

### Effect of degree of relatives in family history on breast cancer features of patients

The effect of degree of relatives with a history of breast cancer on the characteristics of breast cancer is described in Table 4. In our cohort, 776 patients had a family history of breast/ovarian cancer, of which 620 patients had first-degree relatives with breast/ovarian cancer (F-FH), and 156 patients had second-/third-degree relatives with a history of breast/ovarian cancer (S/T-FH). Compared with patients in the S/T-FH group and those without a family history of cancer (median age 46 and 52.3 years, respectively), patients in the F-FH group were significantly older at diagnosis (median age 54.1 years; *p* < 0.001). Compared with patients in the F-FH group and those without a family history of cancer, more patients in the S/T-FH group had stage 0 tumor (ductal carcinoma in situ). The S/F-FH group also had a higher percentage of patients with early disease with N0 and TNM0 stages, and lower percentage of patients with late disease with N2 and TNM-III stages, compared with patients without a family history of cancer. No difference was observed in terms of pathological grade and molecular subtypes between the F-FH, S/T-FH, and non-FH groups.

**Table 4:** Degree of relatives with history of breast/ovarian cancer and patients’ tumor characteristics.

	First-degree relatives (n = 620)		Second-/third-degree relatives (n = 156)		No family history of cancer (n = 7734)		*p*1*	*p*2*	*p*3*
	N	%	N	%	N	%			
**Age at diagnosis (years)**									
Age range	23–88		24–72		18–89		< 0.001^a^	< 0.001^a^	< 0.001^a^
Median age	54.1		46.0		52.3				
**Primary tumor stage**									
T0	38	6.1	23	14.7	550	7.1	< 0.001	0.357	< 0.001
T1	180	29.0	41	26.3	2097	27.1	0.496	0.302	0.817
T2	349	56.3	80	51.3	4463	57.7	0.261	0.492	0.108
T3	46	7.4	8	5.1	506	6.5	0.315	0.398	0.479
T4	7	1.1	4	2.6	118	1.5	0.245	0.434	0.307
**Lymph node stage**									
N0	386	62.3	109	69.9	4667	60.3	0.077	0.348	0.016
N1	146	23.5	32	20.5	1823	23.6	0.420	0.990	0.372
N2	47	7.6	6	3.8	678	8.8	0.098	0.313	0.031
N3	41	6.6	9	5.8	566	7.3	0.701	0.515	0.461
**TNM stage**									
0	38	6.1	23	14.7	546	7.1	< 0.001	0.382	< 0.001^a^
I	129	20.8	29	18.6	1486	19.2	0.539	0.334	0.845
II	350	56.5	83	53.2	4201	54.3	0.466	0.305	0.782
III	102	16.5	20	12.8	1484	19.2	0.265	0.095	0.045
IV	1	0.2	1	0.6	17	0.2	0.362	1.000	0.302
**Histologic grade**									
I	17	2.7	6	3.8	292	3.8	0.435	0.189	0.963
II	465	75.0	110	70.5	5858	75.7	0.253	0.678	0.132
III	138	22.3	40	25.6	1584	20.5	0.369	0.293	0.115
**ER/PR status**									
Positive	450	72.6	116	74.4	5779	74.7	0.655	0.240	0.918
Negative	170	26.4	40	25.6	1955	25.3			
**HER2 status**									
Positive	72	11.6	25	16.0	1102	14.2	0.136	0.069	0.530
Negative	548	88.4	131	84.0	6632	85.8			
**Ki-67 status**									
High	406	65.5	99	63.5	4908	63.5	0.636	0.314	1.000
Low	214	34.5	57	36.5		36.5			
**Subtypes**									
Luminal A	139	22.4	34	21.8	1763	22.8	0.867	0.830	0.768
Luminal B	311	50.2	82	52.6	4016	51.9	0.592	0.397	0.875
HER2	46	7.4	10	6.4	621	8.0	0.663	0.590	0.460
TNBC	124	20.0	30	19.2	1334	17.2	0.830	0.082	0.517

^a^*p* < 0.05 with Tamhane T2 correction. *p1: F-FH vs. S/T-FH. *p*2: F-FH vs. non-FH. *p*3: S/T-FH vs. non-FH.

### Effect of cancer type in family history on breast cancer features of patients

Among the 776 patients, 617 had a family history of only breast/ovarian cancer (single-FH), and 159 patients had a family history of multiple types of cancers (multiple-FH), including breast/ovarian and other types of cancers. Although no significant difference was found in clinical stages and molecular subtypes between single-FH and multiple-FH groups, a 1.5-fold increase in the percentage of patients classified as histologic grade III was observed in the single-FH group (24.6% in single-FH vs. 16.4% in multiple-FH) (Table 5). A similar finding was noted when the single-FH and non-FH groups were compared. Compared with the non-FH group, the single-FH group had a higher percentage of patients with stage N0 (*p* = 0.042), lower percentage of patients with stage TNM-III (*p* = 0.016), but higher percentage of patients with higher-histological-grade tumors (*p* = 0.014) and those with the most aggressive subtype of breast cancer, namely TNBC (*p* = 0.046) (Table 5).

**Table 5:** Types of cancer in relatives and patients’ tumor characteristics.

	Breast/ovarian cancer only (n = 617)		Multiple types of cancer (n = 159)		No family history of cancer (n = 7734)		*p* 1*	*p*2*	*p*3*
	N	%	N	%	N	%			
**Age at diagnosis (years)**									
Age range	23–88		27–79		18–89		0.316	0.697	0.164
Median age	52.2		53.4		52.3				
**Primary tumor stage**									
T0	48	7.8	13	8.2	550	7.1	0.868	0.536	0.606
T1	173	28.0	48	30.2	2097	27.1	0.592	0.619	0.388
T2	343	55.6	86	54.1	4463	57.7	0.734	0.306	0.361
T3	44	7.1	10	6.3	506	6.5	0.710	0.570	0.898
T4	9	1.5	2	1.3	118	1.5	1.000	0.896	1.000
**Lymph node stage**									
N0	398	64.5	97	61.0	4667	60.3	0.413	0.042	0.866
N1	138	22.4	40	25.2	1823	23.6	0.455	0.497	0.641
N2	43	7.0	10	6.3	678	8.8	0.762	0.126	0.273
N3	38	6.2	12	7.5	566	7.3	0.525	0.285	0.913
**TNM stage**									
0	48	7.8	13	8.2	546	7.1	0.868	0.503	0.587
I	123	19.9	35	22.0	1486	19.2	0.562	0.662	0.376
II	350	56.7	83	52.2	4201	54.3	0.306	0.248	0.596
III	94	15.2	28	17.6	1484	19.2	0.463	0.016	0.617
IV	2	0.3	0	0	17	0.2	1.000	0.647	1.000
**Histologic grade**									
I	20	3.2	3	1.9	292	3.8	0.599	0.501	0.214
II	445	72.1	130	81.8	5858	75.7	0.013	0.044	0.079
III	152	24.6	26	16.4	1584	20.5	0.027	0.014	0.201
**ER/PR status**									
Positive	443	71.8	123	77.4	5779	74.7	0.159	0.109	0.449
Negative	174	28.2	36	22.6		25.3			
**HER2 status**									
Positive	83	13.5	14	8.8	1102	14.2	0.114	0.585	0.051
Negative	534	86.5	145	91.2		85.8			
**Ki-67 status**									
High	405	65.6	100	62.9	4908	63.5	0.517	0.279	0.883
Low	212	34.4	59	37.1	2826	36.5			
**Subtypes**									
Luminal A	141	22.9	32	20.1	1763	22.8	0.461	0.974	0.427
Luminal B	302	48.9	91	57.2	4016	51.9	0.062	0.154	0.185
HER2	48	7.8	8	5.0	621	8.0	0.232	0.826	0.167
TNBC	126	20.4	28	17.6	1334	17.2	0.428	0.046	0.905

*p1: single-FH vs. multiple-FH. *p*2: single-FH vs. non-FH. *p*3: multiple-FH vs. non-FH.

### Effect of number of relatives with breast/ovarian cancer on breast cancer features of patients

As previously published, having more relatives with a history of breast/ovarian cancer further increases the risk of developing breast cancer^4^. Among 617 patients with a family history of only breast/ovarian cancer, we further analyzed the relationship between the number of relatives with breast/ovarian cancer and the breast cancer features of the patients (Table 6). In total, 576 patients had only one relative with a history of breast/ovarian cancer, and 41 patients had multiple relatives with a history of breast/ovarian cancer. Patients who had multiple family members with breast/ovarian cancer tended to have higher primary tumor stage and histologic grade, including increased percentage of T3 stage (14.6% vs. 6.6% and 6.5%, *p* = 0.062 and 0.05) and a greater trend of increased percentage of histologic grade III (31.7% vs. 24.1% and 20.5%, *p* > 0.05). Patients who had a single family member with breast/ovarian cancer had a significantly enriched TNBC subtype, compared with patients without a family history of cancer (*p* = 0.048; Table 6).

**Table 6:** Number of relatives with breast/ovarian cancer and patients’ tumor characteristics.

	One relative with B/O cancer history (n = 576)		Multiple relatives with B/O cancer history (n = 41)		No family history of cancer (n = 7734)		*p*1*	*p*2*	*p*3*
	N	%	N	%	N	%			
**Age at diagnosis (years)**									
Age range	23–88		30–79		18–89		0.778	0.325	0.993
Median age	52.2		52.0		52.3				
**Primary tumor stage Primary tumor stage**									
T0	47	8.2	1	2.4	550	7.1	0.357	0.347	0.364
T1	163	28.3	10	24.4	2097	27.1	0.590	0.495	0.709
T2	319	55.4	24	58.5	4463	57.7	0.694	0.276	0.915
T3	38	6.6	6	14.6	506	6.5	0.062	0.959	0.050
T4	9	1.6	0	0	118	1.5	1.000	0.945	1.000
**Lymph node stage**									
N0	369	64.1	29	70.7	4667	60.3	0.389	0.078	0.175
N1	131	22.7	7	17.1	1823	23.6	0.400	0.651	0.328
N2	40	6.9	3	7.3	678	8.8	0.758	0.133	1.000
N3	36	6.3	2	4.9	566	7.3	1.000	0.340	0.767
**TNM stage**									
0	47	8.2	1	2.4	546	7.1	0.357	0.322	0.364
I	113	19.6	10	24.4	1486	19.2	0.400	0.727	0.383
II	327	56.8	23	56.1	4201	54.3	0.758	0.254	0.820
III	87	15.1	7	17.1	1484	19.2	0.735	0.016	0.732
IV	2	0.3	0	0	17	0.2	1.000	0.383	1.000
**Histologic grade**									
I	18	3.1	2	4.9	292	3.8	0.636	0.427	0.668
II	419	72.7	26	63.4	5858	75.7	0.198	0.106	0.066
III	139	24.1	13	31.7	1584	20.5	0.277	0.037	0.076
**ER/PR status**									
Positive	412	71.5	31	75.6	5779	74.7	0.575	0.090	0.896
Negative	164	28.5	10	24.4	1955	25.3			
**HER2 status**									
Positive	77	13.4	6	14.6	1102	14.2	0.818	0.559	0.944
Negative	499	86.6	35	85.4	6632	85.8			
**Ki-67 status**									
High	374	64.9	31	75.6	4908	63.5	0.164	0.479	0.107
Low	202	35.1	10	24.4	2826	36.5			
**Subtypes**									
Luminal A	132	22.9	9	22.0	1763	22.8	0.887	0.947	0.898
Luminal B	280	48.6	22	53.7	4016	51.9	0.532	0.125	0.825
HER2	46	8.0	2	4.9	621	8.0	0.761	0.971	0.770
TNBC	118	20.5	8	19.5	1334	17.2	0.881	0.048	0.702

*p*1: One relative with B/O cancer history vs. Multiple relatives with B/O cancer history. *p*2: One relative with B/O cancer history vs. non-FH. *p*3: Multiple relatives with B/O cancer history vs. non-FH.

### Comprehensive effects of all three aspects of family history

To further analyze the contribution of degree, cancer type, and number of relatives on the breast cancer features of the patients, multiple logistic regression was performed using these three factors (Table 7). We found that compared with patients with a single relative with breast/ovarian cancer, patients with multiple relatives with breast/ovarian cancer had a significantly increased risk of being diagnosed with cancer of a high histologic grade (grade III) (OR 2.08, *p* = 0.048, 95% CI 1.007–4.310). Moreover, compared with the F-FH group, more patients in the S/T-FH group were aged less than 35 years (OR 4.104, *p* < 0.001, 95% CI 2.354–7.155). These three factors did not independently affect other parameters in terms of hormone receptor, HER2, and Ki67 status.

**Table 7:** Multiple logistic regression test on degree, type, and number of relatives with family history of cancer, and patients’ tumor characteristics.

Variable	*p*	OR	95% CI	
			Lower	Upper
**ER**				
Degree	0.763	1.063	0.716	1.576
Type	0.050	1.511	1.001	2.283
Number	0.630	1.183	0.597	2.345
Constant	0.000	2.236		
**HER2**				
Degree	0.224	1.379	0.821	2.315
Type	0.164	0.643	0.345	1.198
Number	0.872	1.076	0.439	2.637
Constant	0.000	0.143		
**Ki67**				
Degree	0.224	1.379	0.821	2.315
Type	0.164	0.643	0.345	1.198
Number	0.872	1.076	0.439	2.637
Constant	0.000	0.143		
**Histologic grade III**				
Degree	0.509	1.185	0.716	1.964
Type	0.110	0.619	0.345	1.114
Number	0.048	2.083	1.007	4.310
Constant	0.000	0.294		
**Age ≤ 35 years**				
Degree	0.000	4.104	2.354	7.155
Type	0.133	0.510	0.212	1.227
Number	0.946	1.044	0.305	3.568
Constant	0.000	0.057		

*Comparison* degree: second-/third-degree versus first-degree relatives, *Type* family history of multiple types of cancer versus breast/ovarian cancer only, *Number* family breast/ovarian cancer history of multiple members versus single member.

## Discussion

Breast cancer is the most commonly diagnosed cancer among women worldwide. Newly diagnosed cases of breast cancer have dramatically increased in the Western world. In addition, breast cancer is becoming a leading cancer in China, accounting for 12.2% of all newly diagnosed cancers^17^. Based on the American Cancer Society, on an average, women have a 13% risk of developing breast cancer in their lifetime^18^. Breast cancer has family aggregation features; about 15% of patients with breast cancer have relatives diagnosed with breast cancer^18^. Family history is one of the major risk factors for developing breast cancer^3^. In this study, we collected data from 10,549 patients with breast cancer, including their tumor features and family history of cancer, and investigated the link between family history and the breast cancer characteristics of the patients.

In our cohort, 7.4% of patients had a family history of breast/ovarian cancer, of which 95.9% patients had a family history of breast cancer only, a percentage that was much higher than that of patients with a family history of ovarian cancer only (3.1%) or that of both cancer types (1%). As the incidence rate of breast cancer (> 100 per 100,000 per year, depends on race, region, etc.) is considerably higher than that of ovarian cancer (11.8 per 100,000 per year)^19,20^, our data cannot rule out the potential inheritable link between family history of ovarian cancer history and breast cancer development. About 19.3% of patients had a family history of other types of cancer, and 73.3% of patients did not have a family history of cancer. The percentage of patients with a family history of breast/ovarian cancer was higher than that reported in another smaller cohort study conducted in the Chinese population by Song et al., which reported 4.84% patients with a family history of breast/ovarian cancer^21^. However, in a study on the Turkish population, 23.5% of patients had a family history of breast/ovarian cancer^22^, and in a study on the European population, 32.7% of patients had a family history of breast cancer^23^. These data suggest that the percentage of patients with a family history of breast/ovarian cancer could depend on the cohort size and population. The percentage of patients with a family history of breast/ovarian cancer in our cohort falls in the normal range.

Another interesting finding from our cohort is the regional aggregation of patients with breast cancer who had a family history of cancer. Significantly higher percentages of patients with family history of breast/ovarian and other types of cancer were found in patients from coastal cities, compared with those in non-coastal cities. The effect of coastal regions on breast cancer is still unclear. Reports by other groups have also shown a higher incidence rate of cervical cancer in coastal regions than in non-coastal regions^24^, which might be attributed to the economic prosperity of coastal cities, leading to better health care and higher diagnosis rate. Moreover, living habits, especially dietary habits, could differ between people living in coastal and non-coastal cities, which could also contribute to the different rates of a family history of breast/ovarian cancer between the two areas.

In our analysis, age at diagnosis was affected the most by having a family history of cancer. We found that patients with a family history of other types of cancer had a slight but significantly higher median age than that of patients with a family history of breast/ovarian cancer or those without a family history of cancer. We also found that patients who had first-degree relatives with a history of breast/ovarian cancer were much more significantly older at diagnosis than patients who had second-/third-degree relatives with a history of breast/ovarian cancer (median age 54.1 vs. 46 years), which is consistent with other published data^22,25^. Besides the age difference, breast cancer tends to be diagnosed early (low primary tumor and TNM stages, similar tumor histologic grade) in patients with second-/third-degree relatives who have breast/ovarian cancer history, compared with patients with first-degree relatives who do not have a breast/ovarian cancer history and patients without a family history of cancer. Current reports mainly focus on the risk of a family history of breast cancer within first- or second-/third- degree relatives, with a breast cancer history of first-degree relatives related to more significantly increased risk of breast cancer^26,27^. To the best of our knowledge, this is among the first reports to analyze the tumor characteristics of patients with a family history of breast cancer within first- or second-/third-degree relatives. This early diagnosis could be linked with the patients’ young age and a family history of breast cancer in their second-/third-degree relatives. With around 8 years of difference in age between them and their relatives, those patients could have better access to medical service and be more educated about breast cancer. The underlying reason of the much younger age at diagnosis in patients with a family history of breast cancer in their second-/third-degree relatives and how or whether their age could contribute to their early diagnosis of breast cancer are still unclear and warrant further investigation.

A family history of breast/ovarian cancer has a significant impact on tumor grade and stage. Patients without a family history of cancer had the highest percentage of histologic grade I tumors (*p* = 0.019); however, they also had a significantly higher percentage of lymph node staging N2 (8.8% vs. 6.8% and 7.3%) and TNM staging III. These data suggest that the breast tumors of patients without a family history of cancer tend to be less aggressive, but the staging at diagnosis tends to be higher than that of patients with a family history of breast/ovarian or other types of cancer. Similar results were found in other studies. In a cohort study published by Jannot et al. on the European population, women who had a family history of breast/ovarian cancer had favorable tumor features and greater overall survival^23^. Another study focused on breast cancer in young women also showed that patients with a family history of breast cancer had smaller tumors and less lymph node positivity at diagnosis^28^. This could be attributed to the possibility that patients who have a family history of cancer are more cautious about their health, leading to early detection of their breast cancers. The degree of relatives that have a history of cancer also impacts the cancer staging of the patient at diagnosis. A significantly lower percentage of T0, N0, and TNM0 staging has been observed in patients with first-degree relatives with a history of breast/ovarian cancer, compared with those with second-/third-degree relatives with a history of breast/ovarian cancer. These data suggest a potential positive effect of the degree of relatives on cancer progression.

The effect of the type of cancer and number of relatives with cancer history on the tumor characteristics of the patients was also analyzed. Increased percentage of tumor histologic grade III was found in patients whose relatives had only breast/ovarian cancer, compared with patients whose relatives had breast/ovarian cancer and other types of cancer, and patients without a family history of cancer. Compared with patients without a family history of cancer, patients with a single relative with breast and ovarian cancer showed higher TNM stage, histologic grade, and TNBC subtype. Patients with multiple relatives with breast and ovarian cancer showed no significant difference from the other two groups. However, owing to the small number of patients during subcategorizing, there were insufficient data to provide a firm conclusion regarding the link between the number of relatives who had breast/ovarian cancer, and the cancer features of the patients.

Although the ER, PR, HER2, and Ki-67 status of the patients’ tumors was not affected by their family history (degree, type, number), the breast cancer molecular subtypes were affected. Patients without a family history of cancer had a slightly increased percentage of luminal B subtype of breast cancer compared with patients without a family history of cancer (51.9% vs. 49.3%, *p* = 0.037). After subcategorizing, compared with patients without a family history of cancer, those with a family history of only breast/ovarian cancer had an increased percentage of TNBC (17.2% vs. 20.4%, *p* = 0.046), and patients with single relatives with a history of breast/ovarian cancer also had an increased percentage of TNBC (17.2% vs. 20.5%, *p* = 0.048).

Family history could influence breast cancer subtypes potentially owing to the enrichment of inherited genetic mutations such as BRCA1 and BRCA2, which account for 30%–50% of the known mutations that cause breast cancer^29,30^. Several studies have reported that breast cancers caused by mutations in BRCA1/2 genes have specific characteristics. A study on 187 patients reported that BRCA1 mutation was associated with TNBC subtype (52.5%), and BRCA2 mutation was associated with luminal B subtype (39.8%)^31^. Another study with a cohort of 425 patients showed similar findings; the most frequently classified subtype for patients with BRCA1 mutation was TNBC (65%), and that for patients with BRCA2 mutation was luminal B subtype (40%)^32^. The increased level of TNBC and luminal B subtypes of breast cancer in patients with a family history of breast cancer in our cohort supported the rationale of enrichment of breast cancer-specific inherited mutations.

One of the major advantages of our study is the large patient cohort. We collected data from 10,549 patients to study the link between family history and breast cancer features. However, as few patients had a family history of breast/ovarian cancer (4–20%, based on literature reports), and owing to our detailed subcategorization to further characterize the degree, type, and number of relatives in breast cancer family history, the number of patients in some groups was too low to draw conclusions. In our future practice, more patient data will be collected to increase the number of patients in each subgroup of patients with a family history of breast/ovarian cancer, which will substantially enhance the strength of our analysis and help strengthen our data.

One limitation of our study is that all the patient cancer history data came from self-reporting of the patients and were not normalized to the patients’ family size. Further, other types of gynecological cancer history can be mistaken for ovarian cancer history during self-reporting by patients. These factors could introduce error to the database. We were unable to analyze the relationship between patient survival and family history of cancer owing to the lack of survival data of patients. We plan to collect patient survival data from this cohort in our future studies. Another long-term study and patient follow-up are currently underway to further investigate the potential role of family history in predicting the progression-free and overall survival of patients. Another limitation of our study is that we did not have genetic mutation testing data for each patient in our cohort, so we could not make direct comparisons of the family history status between patients with or without common mutations. As stated above, considering that we found increased TNBC percentage (around 3%) in patients with a family history of breast/ovarian cancer, and BRCA1 mutation is linked with increased TNBC percentage, common genetic mutations could potentially influence our results. Further in-depth analysis with larger cohort and more family history-related data will be performed in the future.

## Conclusion

After analyzing data from 10,549 patients with breast cancer, we found that patients with a family history of other types of cancer were older at diagnosis than patients with a family history of breast/ovarian cancer or those without a family history of cancer. Patients without a family history of cancer were diagnosed with more advanced disease. Patients with first-degree relatives having breast/ovarian cancer were older and diagnosed at a later stage than patients with second-/third-degree relatives with cancer were. In summary, family history of breast cancer mainly influenced patient age, tumor stage, and grade at diagnosis in our cohort.

## Patients and methods

### Patient data collection

In total, 10,549 patients with breast cancer were recruited from March 2014 to July 2017 in the Department of Breast Surgery, Tianjin Medical University Cancer Institute & Hospital. Data were collected from all patients, including age at diagnosis; family history; TNM staging; histological grade; ER, PR, and HER2 status; and molecular subtypes.

Data regarding family history, including cancer history of relatives within three generations of the patients, were collected at the first visit of the patients. The detailed information was as follows:

1. Degree of relatives. Degree of relatives was classified based on the kinship coefficient. First-degree relatives hold a kinship coefficient of 0.5, i.e., the two individuals have a 50% chance of being genetically identical, including parents, children, and siblings. Second-degree relatives have a kinship coefficient of 0.25, including grandparents, grandchildren, aunt, uncle, niece, nephew, who have a 25% chance of being genetically identical to the patients. Third degree-relatives have a kinship coefficient of 0.125, including half-aunt/uncle, half-niece/nephew, great-grandparents, and great-grandchildren, who have a 1/8th chance of being genetically identical to the patients. The degree of the relatives with cancer history was recorded. Owing to the increased genetic influence with closer relatives, the relatives of patients with cancer history were divided into first-degree and second-/third-degree groups.
2. Cancer types of the relatives. The cancer history of the patients’ relatives was recorded in detail, including their relationship with the patients and the type of primary tumor. If the relatives had multiple types of cancer, only the primary tumor types were recorded, and secondary sites were not included. Owing to the autosomal dominant inheritance of breast/ovarian cancer and the close relationship between these two types of cancer, family history of cancer was further subcategorized into breast/ovarian cancer, other cancer types, and no history of cancer, based on the guidelines for management of hereditary breast and ovarian cancer syndrome published by the American College of Obstetricians and Gynecologists.
3. Number of relatives. The number of relatives who had cancer was recorded, and the patients were sub-categorized into those with a single relative with cancer history and those with multiple relatives with cancer history.

### Pathological and clinical classification

Surgically resected tissues or core biopsy samples that were formalin fixed and paraffin embedded were used for analysis. Cancer staging was performed based on the staging system published by the American Joint Committee on Cancer (AJCC) in 2016. Histologic grading was performed using Black’s method. Breast cancer subtypes were classified by immunohistochemistry (IHC) staining of ER, PR, and HER2. ER or PR was defined as positive when more than 1% tumor cells were positive for either markers. HER2 positive was defined when the tumor IHC score of HER2 was 3+ , or when a 2+ HER2 score with HER2 gene amplification was detected by fluorescence in situ hybridization. Ki-67 high was defined when more than 20% tumor cells were positive for Ki-67 in the primary tumor, and Ki-67 low was defined when less than 20% tumor cells were positive. Breast cancer subtype classification was analyzed following St. Gallen classification of breast cancer 2017^33^. Briefly, luminal A was defined as ER and or PR (≥ 20% positive) positive, HER2 negative, and Ki-67 low. Luminal B was defined as ER and or PR positive, HER2 negative, and Ki-67 high or ER and or PR positive, HER2 positive, and any Ki-67 expression. HER2 positive subtype was defined as ER and PR negative, and HER2 positive. Triple negative breast cancer (TNBC) subtype was defined when ER, PR, and HER2 were all negative in the tumor cells.

### Statistical analysis

All statistical analyses were performed using IBM SPSS 20 software. Non-parametric test was used to analyze the age differences between the patients. The χ^2^ test was used to compare the tumor features (tumor stage, lymph node status, TNM stage, histologic grade, ER, PR, HER2, Ki-67 status, and molecular subtypes) of the patients. Multiple comparison was performed using Tamhane T2, with significant results indicated in the tables. Multiple logistic regression was used to analyze the independent family history variables that contributed to the differences between the tumor features of the patients. Data were considered significant when *p* < 0.05.

### Ethics approval

This study was approved by the ethical committee of Tianjin Medical University Cancer Institute & Hospital (bc2020070). All methods were carried out in accordance with relevant guidelines and regulations.

### Informed consent‬‬‬‬‬‬

Informed consent was obtained from all participants‬.‬‬‬‬‬‬‬‬

## Data Availability

The datasets during and/or analyzed during the current study are available from the corresponding author on reasonable request.

## Abbreviations

BMI: Body mass index
BRCA1: Breast cancer type 1
BRCA2: Breast cancer type 2
HBOC: Hereditary breast and ovarian cancer
NCCN: National Comprehensive Cancer Network
ER: Estrogen receptor
PR: Progesterone receptor
HER2: Human epidermal growth factor receptor 2
AJCC: American Joint Committee on Cancer
IHC: Immunohistochemistry
TNBC: Triple negative breast cancer
FHBO: Family history of breast/ovarian cancer
FHO: Family history of other cancer
Non-FH: No family history of cancer
F-FH: First-degree relatives with breast/ovarian cancer
S/T-FH: Second-/third-degree relatives with breast/ovarian cancer
Single-FH: Family history of only breast/ovarian cancer
Multiple-FH: Family history of multiple types of cancers
